# Foreign Hospitals

**Published:** 1894-11-10

**Authors:** 


### FOREIGN HOSPITALS.

### THE ST. JOSEPH'S HOSPITAL, COPENHAGEN.

This hospital, which owes its origin to the Sisters of St. Joseph—who have their principal domicile at Chamberg in the Savoy—dates from 1875, but this is the first time the hospital has furnished any particulars as to its working. As early as 1856 four sisters came to Copenhagen, where they nursed patients during the first sixteen years in the town and instructed poor children. Thanks to their untiring work a portion of the present hospital was opened in 1875, containing then but 20 beds. In 1881 the west wing was erected, the hospital thereby being enabled to receive 95 patients in 22 single and eight common wards. A convalescent home has since been added, where the patients are received free of charge, and where they can remain several months. The sisters, of whom there are now 30, not only do the nursing but also attend to the office work, &c. The result of their work becomes all the more admirable when one considers that they receive no support from State, corporation, or any public institution. The number of patients received at the hospital since its opening reaches an aggregate of 7,648, the annual average at present being about 850 patients. The average expense per patient per day is 1 Kr. 90 ore, or about 2s. 1d., whilst the large corporation hospitals are worked on a basis of 3s. This considerable difference is no doubt greatly due to the facts that the sisters receive no remuneration, and the expense would probably be lower still did they supply less excellent food than they do. The hospital receives patients of all denominations, and no propaganda for the Roman Church must be carried on within it. The hospital is always in great demand, owing in the first place to the excellent nursing by the sisters, and also to the very moderate charges, viz., 1s. 8d., 2s. 5½d., 3s. 4d., and 4s. 5½d., according to the patient being in a general ward, with first-class fare, in a double or single room, and the charges are the same for patients from Copenhagen or other parts of the country, which, of course, cannot very well be the case at the corporation hospitals. It is a speciality at this hospital that a small number of prominent physicians are at liberty to send their patients and attend to them themselves. There are three doctors attached to the hospital. The sanitary conditions have been exceptionally good, the only instance of hospital diseases being one case of erysipelas during last year.

